# Dynamic clonal evolution of multiple bypass resistance mechanisms in *EGFR*-mutant lung adenocarcinoma: a case report

**DOI:** 10.3389/fonc.2026.1912859

**Published:** 2026-09-09

**Authors:** Qingyun Zhang, Xiaoyan Yin

**Affiliations:** Department of Oncology, Weifang People’s Hospital (The First Affiliated Hospital of Shandong Second Medical University), Weifang, China

**Keywords:** acquired resistance, aumolertinib, clonal evolution, *EGFR* L858R mutation, non-small-cell lung cancer

## Abstract

Third-generation epidermal growth factor receptor (*EGFR*) tyrosine kinase inhibitors (EGFR-TKIs) have markedly improved outcomes for patients with *EGFR*-mutant non-small-cell lung cancer (NSCLC). However, acquired resistance remains a critical clinical challenge.Here, we present the case of a 62-year-old male with stage IIB poorly differentiated lung adenocarcinoma harboring an *EGFR* L858R mutation. The patient received adjuvant aumolertinib following surgical resection. Serial sampling and dynamic next-generation sequencing (NGS) revealed a sequential bypass resistance clone evolution trajectory in this patient: initial *MET* amplification was followed by the detection of a *BRAF* V600E mutation concurrent with *RET* fusion, while in the terminal phase, *MET* amplification re-emerged alongside a progressively increasing *TP53* variant allele frequency (VAF).Genotype-guided therapy adjustments yielded an 11-month progression-free survival (PFS) with aumolertinib plus savolitinib, whereas subsequent selpercatinib combined with chemotherapy provided only transient disease stabilization. Ultimately, the patient died of respiratory and circulatory failure due to progressive disease complicated by diffuse carcinomatous lymphangitis. Based on current evidence, we speculate that these bypass resistance alterations are driven by reactivation of the MAPK and PI3K/AKT signaling cascades. The progressive increase in *TP53* mutant allele frequency was temporally concordant with the sequential emergence of bypass resistance mechanisms, suggesting an unfavorable prognosis. However, its utility in guiding therapeutic modifications requires further validation. This case illustrates that *EGFR* L858R-mutant lung adenocarcinoma can undergo stepwise clonal evolution of multiple bypass resistance mechanisms under aumolertinib selective pressure. Dynamic NGS profiling enables real-time tracking of resistant clone evolution to inform individualized treatment strategies.

## Introduction

Non-small cell lung cancer (NSCLC) accounts for approximately 85% of all lung malignancies, with lung adenocarcinoma representing the most prevalent histological subtype (1, 2). Epidermal growth factor receptor (*EGFR*) mutations constitute a major oncogenic driver in lung adenocarcinoma, occurring in up to 50% of Asian patients (3). The L858R point mutation in *EGFR* exon 21, a canonical activating alteration, accounts for 40%–45% of all *EGFR* mutations and is strongly associated with favorable clinical responses to EGFR-tyrosine kinase inhibitor (EGFR-TKI) therapy (4).

Third-generation EGFR-TKIs, including osimertinib, aumolertinib, and furmonertinib, have emerged as standard first-line and adjuvant treatments for *EGFR*-mutant NSCLC, owing to their high selectivity for mutant *EGFR*, superior antitumor efficacy, and favorable safety profiles (5–8). Aumolertinib, a third-generation EGFR-TKI developed in China, has demonstrated remarkable efficacy in both first-line and adjuvant settings, significantly prolonging progression-free survival (PFS) and overall survival (OS) while improving patients’ quality of life (8).

Nevertheless, despite the substantial clinical benefits of third-generation EGFR-TKIs, acquired resistance remains almost inevitable. In the first-line setting, resistance typically emerges within 18–22 months of treatment initiation, posing a major challenge to achieving favorable long-term outcomes in patients with *EGFR*-mutant NSCLC (9). The mechanisms underlying resistance are highly complex and heterogeneous, encompassing *EGFR*-dependent alterations (e.g., *EGFR* C797S, L792, L718, L798I, E709K, L792V, and G796S/R mutations) and *EGFR*-independent pathways, including activation of downstream or bypass signaling cascades and histological transformation (5).

Among *EGFR*-independent resistance mechanisms, *MET* amplification is the most prevalent event following third-generation EGFR-TKI treatment, occurring in over 15% of patients receiving first-line osimertinib (10). In contrast, activation of other bypass pathways—such as *BRAF* V600E mutation and *RET* fusion—is relatively uncommon, each accounting for less than 5% of resistant cases (10). Notably, the sequential emergence of three distinct bypass resistance mechanisms (*MET* amplification, *BRAF* V600E mutation, and *RET* fusion) in a single patient during acquired resistance to third-generation EGFR-TKIs has not been previously documented.

Tumor clonal evolution is a core hallmark of acquired resistance to targeted therapy, and dynamic shifts in resistance mechanisms directly inform subsequent treatment decisions. Serial biopsy and next-generation sequencing (NGS) at each disease progression are therefore critical for delineating the evolutionary dynamics of resistance and guiding personalized precision therapy (11). Owing to the relative rarity of cases with sequentially arising bypass resistance mechanisms, the evolutionary patterns and optimal clinical management strategies for such patients remain poorly defined.

We present a case of a 62-year-old male patient with *EGFR* L858R-mutant lung adenocarcinoma who developed acquired resistance to postoperative adjuvant aumolertinib, accompanied by the sequential emergence of *MET* amplification, *BRAF* V600E mutation, and *RET* fusion. We further discuss the potential mechanisms underlying clonal evolution and the clinical utility of serial biopsy and longitudinal NGS monitoring, aiming to provide novel insights into the management of acquired resistance to third-generation EGFR-TKIs.

## Case description

**Figure 1:**
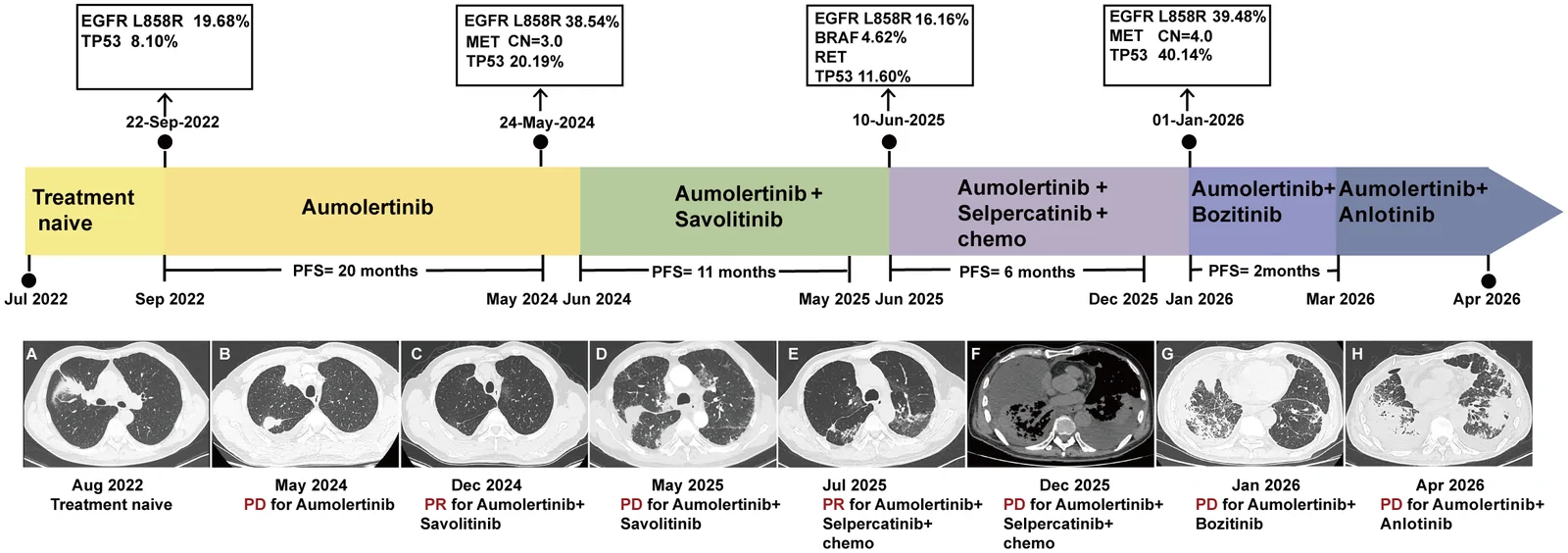
Schematic clinical treatment timeline and sequential molecular evolution of driver gene alterations throughout the disease course.Treatment course and radiological evaluation. The patient was diagnosed in July 2022. **(A)** Baseline CT in August 2022 showed treatment-naive disease. Aumolertinib monotherapy was initiated in September 2022, yielding a progression-free survival (PFS) of 20 months. **(B)** Progressive disease (PD) was documented in May 2024. **(C)**The first-line regimen of aumolertinib plus savolitinib achieved partial response (PR) in December 2024, **(D)** followed by PD in May 2025 (PFS = 11 months). Second-line therapy comprised aumolertinib, selpercatinib, and chemotherapy, **(E)** with PR in July 2025 and **(F)** PD in December 2025 (PFS = 6 months). **(G)** Third-line aumolertinib plus bozitinib resulted in PD after 2 months. Treatment was switched to aumolertinib plus anlotinib in January 2026, **(H)** with PD assessed in April 2026. Molecular evolution. Baseline next-generation sequencing (NGS) on 22 September 2022 detected *EGFR* L858R(variant allele frequency,VAF = 19.68%)and *TP53*(VAF = 8.10%). Upon aumolertinib resistance, NGS on 24 May 2024 detected *EGFR* L858R(VAF = 38.54%), with emergent *MET* amplification (copy number, CN = 3.0) and *TP53*(VAF = 20.19%). After savolitinib resistance, NGS on 10 June 2025 detected *EGFR* L858R(VAF = 16.16%), *MET* amplification was no longer detected, and a newly acquired *BRAF*(VAF = 4.62%) and *RET* fusion were identified, with *TP53* (VAF = 11.60%). Following progression on selpercatinib plus chemotherapy, NGS on 1 January 2026 detected *EGFR* L858R(VAF = 39.48%), *MET* amplification recurred (CN = 4.0), and *TP53* (VAF = 40.14%). The serial chest CT images below demonstrate the dynamic changes in pulmonary lesions throughout the clinical course.

**Figure 2:**
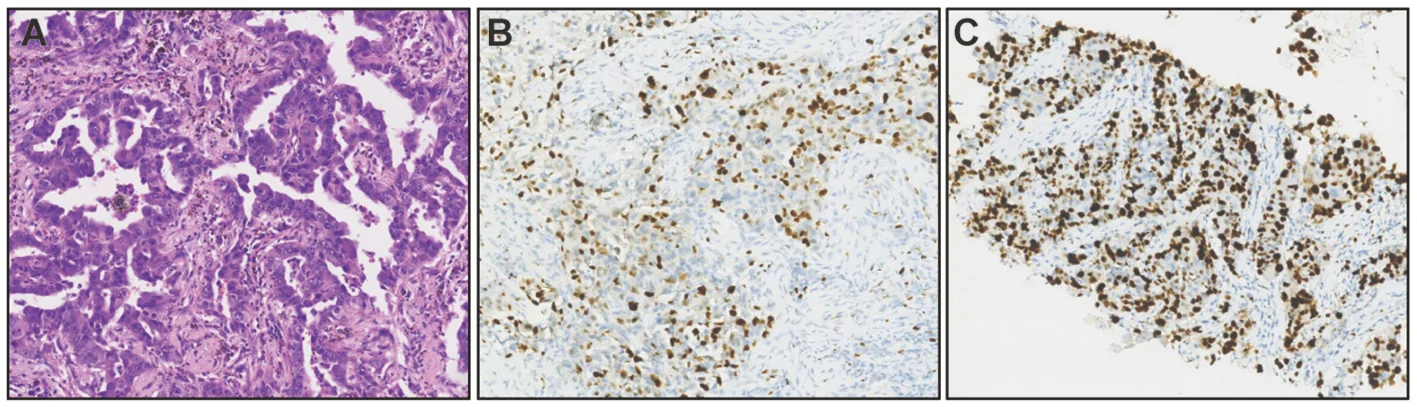
**(A)** Hematoxylin-eosin **(HE)** staining of postoperative tumor tissue (20×). **(B)** Ki-67 labeling index of the primary tumor, approximately 40%.**(C)** Ki-67 labeling index of mediastinal lymph node metastasis, approximately 75%.

A 62-year-old never-smoking male presented with persistent chest tightness and dyspnea in July 2022. Contrast-enhanced chest computed tomography (CT) identified a 4.5 × 2.0 cm solid lesion in the anterior segment of the right upper lobe associated with mediastinal lymphadenopathy (**Figure 1A**). Video-assisted thoracoscopic right upper lobectomy plus systematic lymph node dissection was performed on September 2, 2022.Postoperative pathology verified poorly differentiated invasive lung adenocarcinoma consisting of acinar (65%), solid (30%), micropapillary (4%), and papillary (1%) components (**Figure 2A**). The primary tumor measured 4.5 × 3.0 × 2.0 cm, featuring visceral pleural invasion, bronchial cartilage infiltration, lymphovascular invasion, and metastatic disease in one of two sampled peribronchial lymph nodes. Immunohistochemistry was positive for TTF-1 and Napsin A, with a Ki-67 proliferation index of 40% (**Figure 2B**). Targeted next-generation sequencing (NGS) of resected primary tumor detected activating *EGFR* exon 21 L858R (variant allele frequency, VAF = 19.68%) alongside a somatic *TP53* mutation (VAF = 8.10%). Pathological staging was defined as pT2bN1M0 (stage IIB), and adjuvant oral aumolertinib 110 mg once daily (qd) was administered postoperatively.

The patient was readmitted in May 2024 owing to recurrent chest tightness and dyspnea. Chest CT revealed multiple right-sided pleural metastases (**Figure 1B**), malignant pleural effusion, widespread metastatic lymphadenopathy involving the hilar, mediastinal and supraclavicular regions, and multifocal bone metastases. Ultrasound-guided mediastinal lymph node biopsy confirmed metastatic lung adenocarcinoma with an elevated Ki-67 index of 75% (**Figure 2C**). NGS on metastatic nodal tissue confirmed persistent *EGFR* L858R (VAF = 38.54%), newly acquired *MET* amplification (copy number, CN = 3.0), and elevated *TP53* mutant fraction (VAF = 20.19%). Combination treatment with aumolertinib (110 mg qd) plus savolitinib (400 mg qd) was started in June 2024; concurrent intrapleural cisplatin plus endostar perfusion was applied to control malignant effusion. The patient achieved prominent symptomatic relief and complete effusion resolution, maintaining durable disease control for 11 months (**Figure 1C**).

Disease progression with aggravated chest distress occurred in May 2025, and follow-up CT demonstrated bilateral lymphangitic carcinomatosis and progressive pleural metastases (**Figure 1D**). Serial NGS on pleural effusion-derived tumor cells retained *EGFR* L858R (VAF = 16.16%) and *TP53* mutation (VAF = 11.60%), whereas previous *MET* amplification became undetectable. Two novel druggable genomic alterations were newly identified: *BRAF* V600E (VAF = 4.62%) and CCDC6 exon1-*RET* exon12 in-frame fusion. Treatment was switched to aumolertinib plus selpercatinib 160 mg twice daily (a selective RET tyrosine kinase inhibitor), followed by four cycles of pemetrexed/carboplatin/bevacizumab combination chemotherapy, which yielded only transient disease stabilization (**Figure 1E**).

In December 2025, the patient suffered acute worsening of respiratory symptoms complicated by massive bilateral malignant pleural effusions (**Figure 1F**). Repeat NGS on serially harvested pleural effusion samples showed loss of both *BRAF* V600E and CCDC6-*RET* fusion, accompanied by recurrent *MET* amplification (CN = 4.0) and substantial elevation of *TP53* VAF up to 40.14%. Dual inhibition with bozitinib 200 mg twice daily plus aumolertinib was initiated in January 2026, yet progressive radiographic disease persisted (**Figure 1G**). The regimen was revised to aumolertinib combined with anlotinib 8 mg qd starting on March 5, 2026. Subsequently, the patient’s physical status rapidly declined to Eastern Cooperative Oncology Group performance status (ECOG PS) 3 with refractory cancer cachexia. He was hospitalized emergently for spontaneous pneumothorax on April 2, 2026, and succumbed to combined respiratory and circulatory failure on April 23, 2026 (**Figure 1H**).The whole course of treatment and the sequential molecular evolution of driver gene alterations throughout the disease course are summarized in **Figure 1**.We summarize the detailed clinical course and therapeutic outcome in **Table 1**.

**Table 1:** Clinical course and treatment outcomes under sequential targeted therapies.

Treatment line	Regimen	Time period	Radiographic response	PFS ( months)
Adjuvant Therapy	Aumolertinib 110 mg qd	Sep 2022 – May 2024	NED	20
First-Line Therapy	Aumolertinib 110 mg qd + Savolitinib 400 mg qd Intrapleural perfusion with Cisplatin 40 mg + Endostar 45 mg × 3	Jun 2024 – May 2025	PR	11
Second-Line Therapy	Aumolertinib 110 mg qd + Selpercatinib 160 mg bid Pemetrexed + Carboplatin + Bevacizumab × 4 cycles	Jun 2025 – Dec 2025	SD	6
Third-Line Therapy	Aumolertinib 110 mg qd + Bozitinib 200 mg bid	Jan 2026 – Mar 2026	PD	2
Palliative therapy	Aumolertinib 110 mg qd + Anlotinib 8 mg qd	Mar 2026 – Apr 2026	PD	1

NED, no evidence of disease; PR, partial response; SD, stable disease; PD, progressive disease; PFS, progression-free survival.

## Materials and methods

All NGS testing in this study was performed at Weifang People’s Hospital. Primary tumor tissue, lymph node biopsy, and pleural effusion specimens were analyzed using the Human *EGFR*/*KRAS*/*BRAF*/PIK3CA/*ALK*/ROS1 Gene Mutation Detection Kit (reversible terminator sequencing method; National Medical Device Registration Certificate No. 20213400525; Geneis (Beijing) Co., Ltd.). This panel covers 19 NSCLC-related driver genes, including *EGFR, KRAS, BRAF, PIK3CA, ALK, ROS1, NRAS, HER2, MET, RET, NTRK1/2/3*, and *TP53*. Sequencing was performed on the Illumina MiniSeq platform, with an average target region sequencing depth of ≥ 500×, coverage of ≥ 95%, and a limit of detection for mutant allele frequency of 1%. *MET* amplification positivity was defined as a gene copy number ≥ 1.5.

Tissue and pleural effusion specimens were subjected to identical NGS experimental procedures and bioinformatics analysis pipelines to ensure longitudinal data comparability. Pleural effusion was centrifuged to enrich cell pellets for cell block preparation. Hematoxylin and eosin (HE) staining was performed to assess tumor cellularity, and only samples with sufficient tumor cellularity were subjected to DNA extraction and sequencing. The key variants identified in this case, including the *BRAF* V600E mutation, CCDC6-*RET* fusion, and recurrent *MET* amplification, were all derived from pleural effusion cell block specimens that had passed pathological quality assessment. Previous studies have demonstrated that malignant pleural effusion cell blocks represent reliable alternative specimens for dynamic molecular testing in advanced lung adenocarcinoma, particularly in patients for whom repeated invasive tissue re-biopsy is not feasible (12).

## Discussion

This case, characterized by serial biopsies and dynamic NGS profiling, delineates the stepwise evolution of resistance mechanisms in *EGFR* L858R-mutant lung adenocarcinoma under aumolertinib pressure. Contrary to the relatively uniform patterns of resistance clonal evolution described in previous studies (13, 14), the patient acquired three molecularly distinct bypass-driven resistance mechanisms under persistent targeted selective pressure: *MET* amplification (membrane receptor alteration), *BRAF* V600E (intracellular kinase mutation), and *RET* fusion (chromosomal rearrangement). Of note, *MET* amplification recurred after initial suppression by matched targeted agents, indicative of ongoing dynamic competition between heterogeneous tumor subclones. In addition, longitudinal monitoring of *TP53* mutational kinetics in this case further validates its correlation with unfavorable clinical prognosis.

Dual selective pressure exerted by EGFR-TKI treatment and tumor microenvironment drives tumor clonal evolution, indicating that acquired resistance in *EGFR*-mutant non-small cell lung cancer (NSCLC) does not arise from a single genetic event but rather constitutes a continuous, dynamic evolutionary process (15). We hypothesize that low-abundance resistant subclones pre-existed at baseline but remained undetectable below the NGS limit of detection. During approximately 20 months of aumolertinib monotherapy, *MET*-amplified subclones underwent selective clonal expansion and became dominant, driving initial disease progression. Subsequent combination treatment with savolitinib efficiently suppressed this subclone, yielding 11 months of disease stabilization. Under persistent therapeutic selective pressure, subclones carrying *BRAF* V600E and *RET* fusion acquired a proliferative competitive advantage, driving secondary resistance. Subsequent triple combination therapy comprising aumolertinib, selpercatinib, and chemotherapy achieved only transient disease control. Serial pleural effusion NGS testing revealed concurrent undetectability of *BRAF* V600E and *RET* fusion, which may be explained by treatment-induced clonal suppression, intrinsic liquid biopsy sampling heterogeneity, or technical detection limitations. Thereafter, *RET* fusion became undetectable while *MET* amplification re-emerged, demonstrating dynamic clonal competition and phenotypic switching among heterogeneous resistant subclones under sequential therapeutic selective pressure. Nevertheless, considering the inherent limitations of localized single-site sampling, this clonal evolutionary inference should be interpreted with caution.

All resistance alterations detected in this case were typical bypass activation events, ultimately converging on the reactivation of two core downstream signaling axes: PI3K/AKT and MAPK/ERK. Mechanistically, *MET* amplification sustains tumor cell survival via the ERBB3/PI3K/AKT cascade (16); the *BRAF* V600E mutation drives persistent activation of MAPK signaling (17); and *RET* fusion concurrently activates both MAPK and PI3K/AKT pathways (18). These findings confirm that *EGFR*-mutant lung cancer is highly reliant on these two core signaling networks. Under the selective pressure of *EGFR* inhibition, genomic aberration capable of restoring oncogenic downstream signaling can be selected and become a dominant resistance driver.

Sustained inhibition of the primary *EGFR* pathway combined with concurrent blockade of bypass signaling represents an effective strategy to prolong disease control (19). Savolitinib is the first domestically approved selective MET-TKI in China. It demonstrates favorable efficacy and safety in combination regimens for patients with *MET*-amplified NSCLC who have progressed after prior EGFR-TKI treatment. Accordingly,This patient received aumolertinib plus savolitinib (400 mg once daily), yielding an 11-month progression-free survival (PFS). The 400 mg daily dose was chosen to mitigate the risk of overlapping toxicities associated with dual targeted therapy. Asian subgroup data from the TATTON trial indicated that savolitinib 600 mg once daily (either as monotherapy or combination therapy) induced dose-limiting toxicities (DLTs) in 75% of enrolled patients. By comparison, the 400 mg once daily regimen achieved comparable antitumor efficacy without any grade ≥ 3 DLTs (20). Adverse events were evaluated every 3 weeks per the Common Terminology Criteria for Adverse Events (CTCAE) version 5.0, with routine laboratory surveillance including liver function tests, complete blood count, and QTc interval measurement. This combination regimen demonstrated favorable tolerability, and no grade ≥ 3 adverse events necessitated dose modification.Upon second disease progression, NGS testing identified concurrent *BRAF* V600E (VAF = 4.62%) and *RET* fusion alterations. Selpercatinib has yielded objective response rates ranging from 62% to 83% and a median progression-free survival of 22-26.2 months in patients with *RET* fusion-positive NSCLC (21).In contrast, the combination of dabrafenib plus trametinib (dual BRAF/MEK inhibition) alongside an EGFR-TKI for acquired *BRAF* V600E-driven resistance has only been documented in isolated case reports and small retrospective cohorts (22). Additionally, the relatively low VAF of the *BRAF* V600E mutation further supported prioritization of selpercatinib combined with aumolertinib. Small-cohort clinical evidence has also revealed that triple therapy consisting of dabrafenib, trametinib and osimertinib carries a 57% incidence of pyrexia; multiple patients required treatment interruption or dose de-escalation secondary to intolerable adverse reactions (23). At the time of second progression, the patient presented with an ECOG performance status of 2-3, complicated by concurrent carcinomatous lymphangitis and pulmonary infection, indicating that the risk of severe treatment-related toxicities would likely outweigh potential clinical benefits. Restrictions stemming from the patient’s extensive prior treatment lines and poor performance status rendered the patient ineligible for relevant clinical trials. Ultimately, the dual-targeted regimen was selected after comprehensive discussion with the patient and their family in accordance with their treatment preferences.The subsequent regimen consisting of aumolertinib, selpercatinib, and chemotherapy failed to fully abrogate oncogenic signaling, thereby resulting in limited therapeutic efficacy and rapid disease progression.

Current evidence recognizes *TP53* mutations as an independent marker of poor prognosis (24), with no direct evidence demonstrating their role in driving EGFR-TKI resistance. In this case, the *TP53* VAF demonstrated a progressive increase. Although this measurement is potentially confounded by technical factors including tumor purity, gene copy number variation, and sampling-site heterogeneity, this dynamic trend was temporally concordant with the sequential emergence of bypass resistance mechanisms and radiological disease progression, suggesting that it may reflect clonal expansion. We hypothesize that this is related to loss of *TP53* function and consequent elevated genomic instability (25). Importantly, the direct cause of targeted therapy failure in this patient was bypass activation mediated by *MET* amplification, *BRAF* V600E, and *RET* fusion. The elevated *TP53* mutant allele frequency is biologically linked to an enhanced malignant phenotype (26, 27). The patient’s increased Ki-67 proliferation index and terminal carcinomatous lymphangitis may be associated with *TP53*-related genomic instability, although the specific causal mechanisms warrant further investigation. In summary, dynamic clonal evolution driven by multiple bypass pathway activations constitutes the core resistance mechanism in this case. The dynamic evolution of *TP53* mutations carries prognostic alert value; an increase in mutant allele frequency may indicate an impending phase of rapid disease progression and could potentially inform adjustments to treatment intensity and combinatorial regimens, although prospective evidence is currently lacking.

Serial dynamic NGS testing after disease progression is indispensable for tracking tumor clonal evolution and defining real-time resistance profiles (28). Nevertheless, repeated invasive tissue sampling is not universally clinically feasible. Accordingly, molecular re-assessment should be performed upon disease progression using readily available tumor tissues or validated surrogate biospecimens (e.g., pleural effusion cell blocks, circulating tumor DNA) whenever achievable. This strategy enables timely identification of targetable resistance alterations to guide subsequent individualized therapeutic regimens. Such testing empowers clinicians to uncover precision therapeutic options amid intricate resistance clonal landscapes; however, its clinical implementation requires comprehensive integration of clinical status, patient treatment preferences, and specimen availability. In the present case, NGS profiling-guided personalized therapy delivered successive periods of symptomatic alleviation and incremental survival benefits for the patient.However, persistent disease progression eventually occurred due to intrinsic tumor heterogeneity, compensatory crosstalk among signaling pathways, progressive genomic instability, and continuous decline in the patient’s performance status. This scenario highlights the major clinical challenge of achieving long-term disease control in patients with advanced *EGFR*-mutant NSCLC who develop multiple bypass resistance mechanisms after sequential lines of treatment.

Although the present study delineates a complex clonal evolutionary trajectory of *EGFR*-mutant lung cancer under continuous targeted therapeutic pressure, several limitations should be noted. First, as a single-case study, this work cannot establish causal links between discrete resistance events, and its findings are not generalizable to the overall *EGFR*-mutant non-small cell lung cancer population. Second, genomic data were obtained from surgical specimens, needle biopsy tissues, and pleural effusion samples via NGS testing, which inherently carries potential sampling bias. Most notably, no *in vitro* or *in vivo* functional validation was performed to verify the underlying resistance mechanisms, and neither single-cell sequencing nor phylogenetic evolutionary analysis was conducted. Accordingly, our inferences regarding subclonal competition, suppression, and re-emergence remain hypothetical and cannot provide definitive evidence for tumor evolutionary patterns. In addition, whether these distinct resistant subclones pre-existed at baseline or independently evolved under therapeutic selective pressure remains unclear. Finally, the absence of serial longitudinal circulating tumor DNA (ctDNA) monitoring precluded noninvasive real-time surveillance of the dynamic expansion and contraction of resistant subclones over treatment courses. In conclusion, future prospective, multi-case mechanistic studies integrating single-cell sequencing and dynamic ctDNA surveillance are warranted to further validate the tumor evolutionary model proposed in this study.

## Conclusion

This case uniquely documents the stepwise development of three distinct bypass-driven resistance alterations—*MET* amplification, *BRAF* V600E mutation, and *RET* fusion—in *EGFR* L858R-mutant lung adenocarcinoma following the onset of acquired resistance to aumolertinib, accompanied by dynamic shifts in *TP53* mutant allele abundance. Mechanistically, these bypass aberrations sustain tumor cell viability via reactivation of the MAPK and PI3K/AKT signaling cascades. The progressive rise in the *TP53* variant allele frequency was temporally concordant with radiological disease progression and confers unfavorable prognostic significance; nonetheless, prospective clinical data are still required to validate its value for guiding treatment regimen adjustments. Furthermore, this case highlights the critical importance of repeat molecular reassessment upon disease progression. Given the continuously evolving resistance clonal landscape, NGS profiling using readily accessible alternative biospecimens yields clinically actionable molecular evidence to guide therapy selection, even when serial invasive tissue biopsies are clinically unfeasible.

## Data Availability

The original contributions presented in the study are included in the article/supplementary material. Further inquiries can be directed to the corresponding authors.
